# Role of Volume Replacement during Neonatal Resuscitation in the Delivery Room

**DOI:** 10.3390/children9101484

**Published:** 2022-09-28

**Authors:** Deepika Sankaran, Emily C. A. Lane, Rebecca Valdez, Amy L. Lesneski, Satyan Lakshminrusimha

**Affiliations:** 1 Department of Pediatrics, University of California, Davis, Sacramento, CA 95817, USA; 2 Department of Stem Cell Research, University of California, Davis, Sacramento, CA 95817, USA

**Keywords:** epinephrine, flush volume, neonatal resuscitation, chest compressions, asphyxia, cardiac arrest newborn, hypovolemia, volume bolus

## Abstract

Volume expanders are indicated in the delivery room when an asphyxiated neonate is not responding to the steps of neonatal resuscitation and has signs of shock or a history of acute blood loss. Fetal blood loss (e.g., feto-maternal hemorrhage) may contribute to perinatal asphyxia. Cord compression or a tight nuchal cord can selectively occlude a thin-walled umbilical vein, resulting in feto-placental transfusion and neonatal hypovolemia. For severe bradycardia or cardiac arrest secondary to fetal blood loss, Neonatal Resuscitation Program (NRP) recommends intravenous volume expanders (crystalloids such as normal saline or packed red blood cells) infused over 5 to 10 min. Failure to recognize hypovolemia and subsequent delay in volume replacement may result in unsuccessful resuscitation due to lack of adequate cardiac preload. However, excess volume load in the presence of myocardial dysfunction from hypoxic–ischemic injury may precipitate pulmonary edema and intraventricular hemorrhage (especially in preterm infants). Emergent circumstances and ethical concerns preclude the performance of prospective clinical studies evaluating volume replacement during neonatal resuscitation. Translational studies, observational data from registries and clinical trials are needed to investigate and understand the role of volume replacement in the delivery room in term and preterm neonates. This article is a narrative review of the causes and consequences of acute fetal blood loss and available evidence on volume replacement during neonatal resuscitation of asphyxiated neonates.

## 1. Introduction

Most neonates transition to extrauterine life without intervention and require minimal to no assistance to breathe at birth [1], while ~1% require advanced resuscitation including chest compressions [2]. Approximately half of neonates receiving chest compressions in the delivery room require epinephrine [2]. Neonates with perinatal asphyxia who survive after extensive resuscitation are at risk of neurodevelopmental disability [3]. Impaired placental functions resulting in hypoxia and fetal blood loss (e.g., feto-maternal hemorrhage) are common etiologies of perinatal asphyxia. Neonatal shock is a state of acute circulatory failure with inadequate tissue perfusion, and is commonly caused by asphyxial insult, fetal blood loss or sepsis [4]. Volume replacement for neonates in the delivery room remains a knowledge gap for neonatal providers [5,6]. It is difficult to distinguish euvolemic infants with asphyxia from those with hypovolemic or hemorrhagic shock (e.g., occult blood loss) in the delivery room [7]. While not recommended for asphyxia without blood loss (i.e., euvolemic asphyxia), early rapid volume replacement may be of benefit in improving outcomes in infants with hypovolemia in the delivery room.

## 2. Discussion

### 2.1. Current American Recommendations on Volume Replacement in the Delivery Room

The current (8th) edition of the *American Academy of Pediatrics—Neonatal Resuscitation Program (NRP)* textbook recommends administration of a volume expander if the neonate is not responding to initial steps of resuscitation and there are signs of shock or a history of acute blood loss [1]. For severe bradycardia or cardiac arrest associated with fetal blood loss that does not respond to initial cardiopulmonary resuscitation (CPR), intravenous epinephrine is administered, with the subsequent use of volume expanders (crystalloid or red blood cells). Crystalloids such as normal saline or emergency-release O-negative blood are infused over 5 to 10 min. Current guidelines are based on expert opinion (level of evidence C-EO (expert opinion), “moderate” class of recommendation, or benefits outweigh risks) [8]. More research is needed to identify indications and timing of volume replacement during neonatal resuscitation. Questions as to the type and amount of volume expander, rate of infusion, and assessment of response to volume replacement remain unanswered.

### 2.2. Clinical Suspicion of Hypovolemia due to Blood Loss

**Figure 1:**
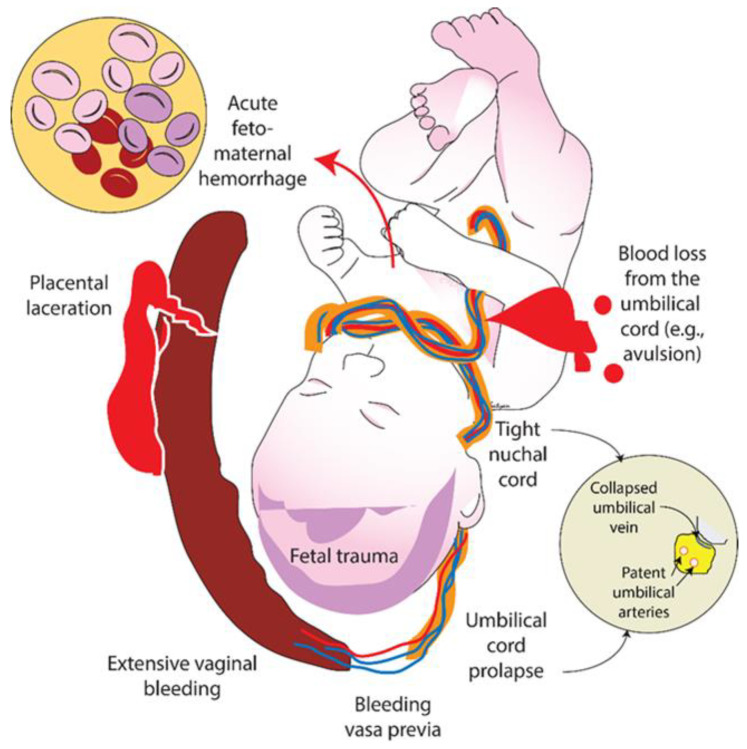
Acute blood loss may be secondary to various causes listed in the illustration. In infants born through a tight nuchal cord, there may be partial umbilical cord occlusion compressing the collapsible umbilical vein decreasing blood flow into the fetus but allowing blood flow out of the fetus into the placenta through the umbilical arteries (inset). Copyright Satyan Lakshminrusimha.

Neonatal shock is a state of acute circulatory failure with inadequate tissue perfusion. It is commonly caused by an asphyxial insult, partial cord compression, fetal blood loss or sepsis [4]. If delivery is complicated by acute feto-maternal hemorrhage, feto-placental bleed, bleeding vasa previa, extensive vaginal bleeding, a placental laceration, fetal trauma, umbilical cord prolapse, tight nuchal cord, or blood loss from the umbilical cord, the neonate might be in hypovolemic shock (Figure 1).

**Figure 2:**
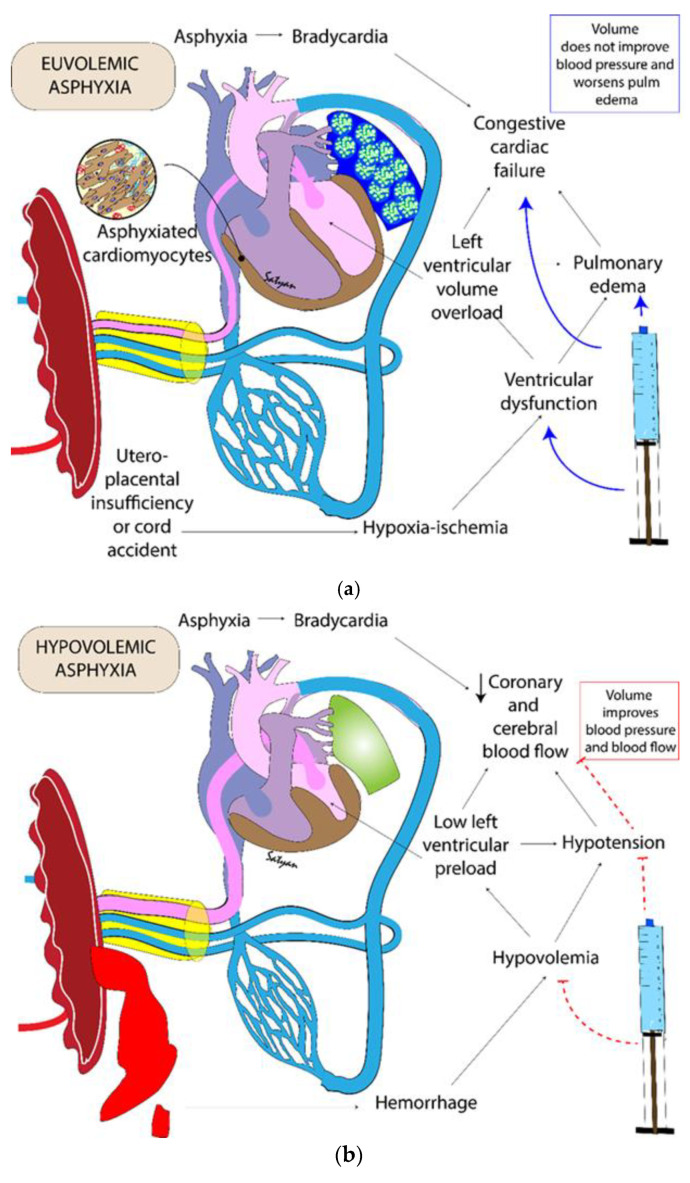
Illustration of euvolemic asphyxia (**a**) and hypovolemic asphyxia (**b**) and potential consequences of volume replacement during resuscitation. Copyright Satyan Lakshminrusimha.

In hypovolemic cardiac arrest secondary to fetal blood loss, there is a decrease in preload to the heart due to a decrease in circulating blood volume. Neonatal cardiac output is the product of stroke volume and heart rate, where the stroke volume is determined by the left ventricular preload, cardiac contractility, and afterload. The immature myocardium in an asphyxiated neonate is unable to increase the intrinsic cardiac contractility due to depletion of energy reserves [9]. Thus, cardiac output is dependent on adequate left ventricular preload and heart rate. In bradycardia induced by hypovolemic asphyxia, both preload and heart rate are reduced, severely limiting cardiac output (Figure 2). We speculate that, in euvolemic asphyxia, volume replacement during resuscitation may lead to left ventricular volume overload, pulmonary edema, and congestive cardiac failure due to left ventricular dysfunction (secondary to asphyxiated cardiomyocytes) (Figure 2a). In contrast, we speculate that volume replacement may potentially improve left ventricular preload, coronary and cerebral blood flow in hypovolemic asphyxia (Figure 2b).

The current American guidelines [1] for neonatal CPR recommend positive pressure ventilation and chest compressions followed by intravenous epinephrine [1]. Typically, volume replacement is administered after epinephrine if blood loss is suspected and there is no response to epinephrine. We speculate that such an approach may be appropriate in normovolemic asphyxial arrest or if fetal blood loss is minimal to result in return of spontaneous circulation (ROSC). In the case of hypovolemic cardiac arrest with moderate to severe fetal blood loss, however, this approach may not be sufficient to allow adequate coronary and cerebral blood flow. Furthermore, chest compressions and epinephrine may be less effective in increasing the diastolic blood pressure (as coronary perfusion mainly occurs during diastole) due to lack of adequate preload. An intravenous normal saline bolus during CPR may quickly replenish the left ventricular preload, enhancing the effectiveness of chest compressions and intravenous epinephrine to potentially expedite ROSC.

A neonate born after acute fetal blood loss may have a persistently low heart rate, appear pale, have delayed capillary refill and/or have weak pulses [3]. In some cases, there will be signs of shock with no obvious evidence of blood loss. Administration of a volume expander is indicated if the asphyxiated neonate is not responding to the steps of resuscitation and there are signs of shock or a history of acute blood loss [1]. However, this approach may be challenging in the absence of a history of blood loss due to difficulty in differentiating the signs of shock from euvolemic asphyxia in the delivery room. Secondly, when hypovolemic shock is suspected based on sinusoidal fetal heart tracing, or low hemoglobin on a blood gas sample, we speculate that end-organ damage may have occurred, and volume replacement after detection of these signs during CPR may delay ROSC or lead to neurological morbidity.

Clinical assessments of perfusion may help to differentiate between euvolemic and hypovolemic states. However, evaluations of color of mucus membranes, capillary refill time, color of infant, palpation of peripheral pulses, are all subjective and prone for inter-observer variability [10]. Based on evaluation of available evidence, the Neonatal Life Support (NLS) taskforce concluded that “early volume replacement with crystalloid or red cells is indicated for neonates with blood loss who are not responding to resuscitation” [5]. There is insufficient evidence to support routine volume replacement in neonates without blood loss who is refractory to ventilation, chest compressions, and epinephrine. However, as blood loss may be occult, a trial of volume replacement may be considered in those infants not responding to resuscitation. Caution should be exercised, as giving a large volume load to a heart injured by asphyxia may actually worsen cardiac output, further compromising the newly born infant (Figure 2a) [1].

### 2.3. How Does the Fetus Respond to Hypovolemia?

The fetoplacental circulation consists of circulating blood volume of about 120–135 mL/kg fetal body weight [11]. Animal studies were performed to investigate the fetal response to hypovolemia secondary to acute blood loss from fetoplacental circulation. Robillard et al. instrumented 11 fetal lambs at 103–138 days gestation in utero (term gestation being 147 to 150 days), and catheters were placed in the fetal femoral artery and vein and superficial tributary of the maternal femoral artery [12]. Following removal of fetal blood (induced fetal hemorrhage) in a stepwise fashion of about 25–35% of fetoplacental blood volume in aliquots of 25 mL every 10 min, there was a decrease in fetal hematocrit (34.7 ± 2.58 to 27.0 ± 1.6), pH (7.38 ± 0.01 to 7.35 ± 0.01), mean arterial blood pressure (58.1 ± 2.59 to 52.2 ± 2.6 mmHg) and plasma proteins (3.14 ± 0.15 to 2.78 ± 0.19 g/dL). Simultaneously, there was an increase in fetal plasma arginine vasopressin from 0.73 ± 0.21 to 34.9 ± 10.04 pU/mL (*p* < 0.01) and fetal plasma renin activity from 4.78 ± 2.22 to 40.4 ± 18.31 ng/mL/h (*p* < 0.05). The rise in fetal plasma arginine vasopressin appeared when the fetoplacental blood loss was between 6 and 10%. This demonstrates that a significant reduction in fetal blood volume occurs prior to stimulation of posterior pituitary to release arginine vasopressin. Once the fetoplacental blood volume was corrected by transfusion, all the plasma vasopressin values returned to baseline within 2 h. However, the fetal plasma electrolytes (sodium, potassium, and chloride) and osmolality remained stable during the fetal hemorrhage and 2 h after the blood volume was corrected by transfusion. With fetal hemorrhage, the fetal heart rate increased from 183 ± 9.86 to 205 ± 12.48/min but was not statistically significant. The authors suggest that, following fetal blood loss, there are isosmotic water fluxes from the fetal interstitial space to fetal vascular compartment to compensate for the blood loss. Additionally, the increase in fetal plasma arginine vasopressin (or anti-diuretic hormone), which is a potent systemic vasoconstrictor, results in an increase in the systemic vascular resistance and fetal mean arterial blood pressure. Following bleeding of fetal goats, Zanjani et al. observed an increase in fetal plasma erythropoietin concentrations [13]. Additionally, they observed an increase in maternal and fetal erythropoietin concentrations with bleeding of pregnant goats. This production of fetal erythropoietin appears to be driven by the relative availability of oxygen to the fetal tissues.

### 2.4. Evidence on Volume Replacement in Asphyxiated Neonates from Animal Studies

The left ventricular stroke volume increases significantly after volume infusion in term and preterm lambs [14]. A randomized trial in postnatal piglets with euvolemic asphyxia induced by ventilation at low rates with a gas mixture of 7.5% CO_2_ and 5.3% O_2_, compared 5% albumin, normal saline, and no volume infusion (sham) on development of pulmonary edema and restoration of mean arterial pressure (MAP) during resuscitation of asphyxiated piglets (Table 1) [15]. At 2 h post-resuscitation, MAP of sham (48 ± 13 mm Hg) and albumin groups (43 ± 19 mm Hg) was higher than normal saline group (29 ± 10 mm Hg; *p* = 0.003 and 0.023, respectively). After resuscitation, sham piglets had less evidence of pulmonary edema (wet to dry lung weight ratio: 5.84 ± 0.12 vs. 5.98 ± 0.19; *p* = 0.03) and better dynamic lung compliance (Cd) compared to albumin or normal saline groups (Cd: 1.43 ± 0.69 vs. 0.97 ± 0.37 mL/cm H_2_O, *p* = 0.018). Volume infusion during resuscitation did not improve MAP, and acute recovery of MAP was poorer with normal saline compared with albumin groups. Volume infusion was associated with increased pulmonary edema. The authors concluded that in the absence of hypovolemia, volume infusion during neonatal resuscitation is not beneficial.

A randomized trial in a postnatal piglet model of hypovolemic cardiac arrest reported no differences in ROSC outcomes with saline vs. blood transfusion used for volume replacement (Table 1) [16]. It is not clear whether volume expanders make a difference in asphyxia induced by umbilical cord compression which resembles clinical conditions such as nuchal cord and umbilical cord prolapse. In these conditions, it is possible that compression of the thin-walled umbilical vein occurs early compared to compression of the thick-walled high-pressure umbilical arteries (Figure 1—inset). However, there are no randomized studies comparing no volume replacement vs. volume expanders in neonatal CPR in hypovolemic cardiac arrest in a perinatal model.

**Figure 3:**
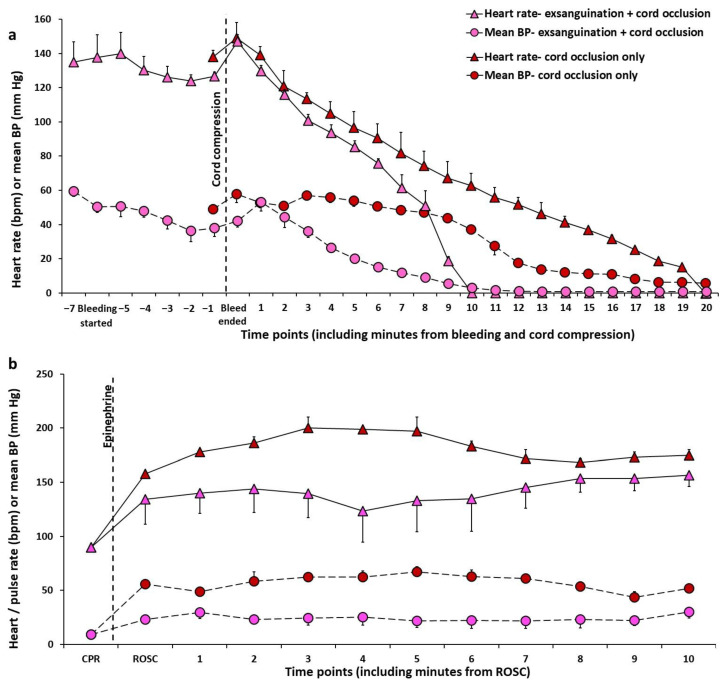
Changes in heart rate (beats per min, bpm) and mean blood pressure (mean BP, mm Hg) during exsanguination followed by cord occlusion in an ovine model (**a**,**b**), and during asphyxia by cord occlusion (**a**,**b**). The heart rate and mean BP decrease quickly and dramatically in hypovolemic cardiac arrest (**a**) with slower return to baseline after return of spontaneous circulation (**b**). Copyright Deepika Sankaran.

**Figure 4:**
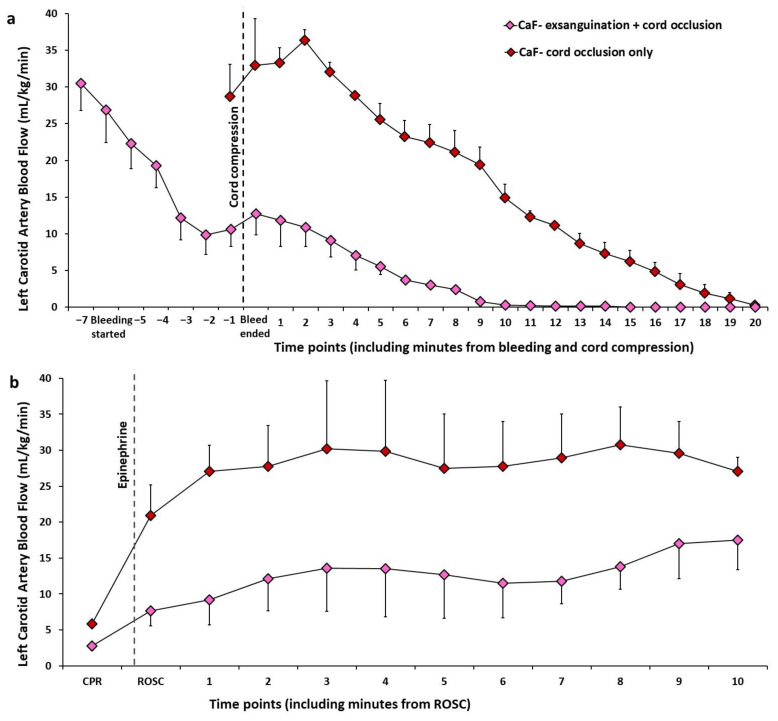
Changes in left carotid artery blood flow (CaF, mL/kg/min) during exsanguination followed by cord occlusion and during asphyxia by cord occlusion alone. The CaF decreases quickly and drastically during exsanguination in hypovolemic cardiac arrest (**a**). Following return of spontaneous circulation (ROSC), there is slower recovery of CaF in hypovolemic cardiac arrest when compared to normovolemic cardiac arrest (**b**). Copyright Deepika Sankaran.

*Validation of an Ovine Model of Hypovolemic Asphyxial Arrest:* To validate the hypovolemic asphyxial cardiac arrest model, we compared hemodynamics during asphyxia with cord occlusion alone in two fetal lambs and during exsanguination (removal of 45 mL/kg of blood from jugular venous catheter) followed by cord occlusion in three other fetal lambs (Figure 3 and Figure 4). These experiments were performed by the authors (D.S., S.L., A.L.L., E.C.A.L. and R.V.) after obtaining approval from the Institutional Animal Care and Use Committee (IACUC) at University of California at Davis and include unpublished data from ongoing studies evaluating the role of volume replacement during resuscitation in neonatal hypovolemic cardiac arrest.

In these studies, following intubation and induction of general anesthesia in time-dated pregnant ewes, fetal lambs were partially exteriorized, and lines were placed in the right carotid artery and jugular vein. A flow probe was placed around the left carotid artery. Heart rate, blood pressure and carotid flow were continuously monitored. One group of lambs induced fetal blood loss by drawing 45 mL/kg of arterial blood from the carotid line and the second group had lines placed without any blood loss. Subsequently, both groups of lambs underwent asphyxia by umbilical cord occlusion. We observed that blood loss hastened bradycardia, hypotension, left carotid arterial blood flow, and cardiac arrest in the hypovolemic compared to normovolemic lambs. The mean left carotid arterial blood flow decreased drastically (30.5 ± 3.7 to 10.6 ± 2.3 mL/kg/min) at the end of exsanguination although the heart rate and blood pressure did not significantly change with exsanguination when compared to baseline (Figure 3 and Figure 4). These findings suggest that fetal heart rate may not be a reliable measure of moderate fetal volume loss.

Moreover, there was slow recovery of heart rate, mean blood pressure and left carotid artery blood flow after ROSC among the lambs in hypovolemic cardiac arrest (Figure 3 and Figure 4).

We have previously shown that a larger flush volume following IV epinephrine via a low umbilical venous catheter increases the incidence of and expedites ROSC in ovine neonatal asphyxial arrest but this may be secondary to better delivery of the medication [17,18]. No conclusive evidence regarding the role of volume replacement on epinephrine effectiveness can be derived from this study.

### 2.5. Evidence on Volume Replacement from Clinical Studies

There is limited evidence available from human studies. In a retrospective review of 37,972 infants over a 30-month study period from a resuscitation registry that was maintained in a single center (Parkland Memorial Hospital, Dallas, TX, USA) with large delivery volume (>15,000 deliveries annually), 13 out of 23 infants (57%) who received chest compressions were treated with volume infusion in the delivery room, and only 3 of those infants had clinical suspicion of hypovolemia (history of increased risk of fetal blood loss) prior to volume replacement [7]. Infants who received volume infusion in the delivery room exhibited more fetal heart rate abnormalities during labor, more severe fetal and neonatal acidemia, lower Apgar scores at 5 and 10 min, and lower blood pressures on admission to the NICU and during the first 2 postnatal hours, compared to infants who did not receive volume infusion. It is possible that the sicker babies received volume infusion and hence had poorer outcomes.

In hemodynamically stable preterm infants born at ≤32 weeks gestation, a Cochrane review did not find any benefit of early volume replacement [19]. A systematic review by Keir et al. identified two studies on volume replacement, with only one in the delivery room, and suggested that volume replacement should be reserved for acute hemorrhage (Table 2) [20]. A multicenter observational cross-sectional survey by Keir et al. included 5 out of 163 neonates who received a fluid bolus during resuscitation, with no further details provided [21].

### 2.6. Potential Harm from Volume Replacement in the Delivery Room

**Figure 5:**
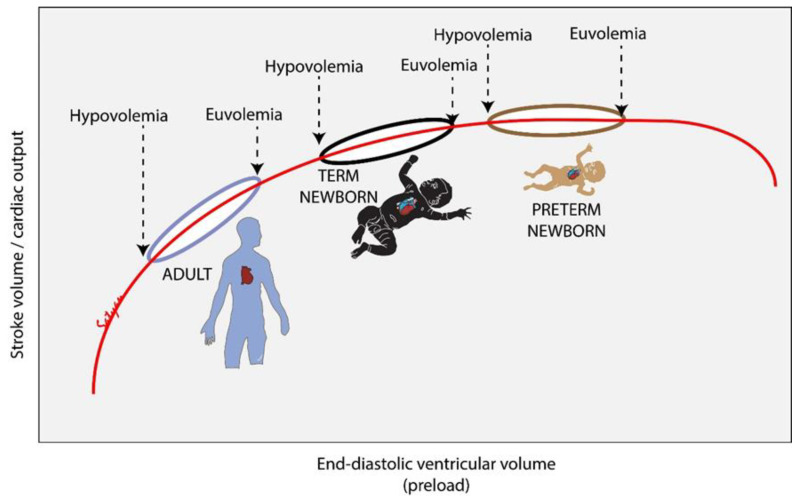
Change in stroke volume/cardiac output with change in end-diastolic ventricular volume (Frank–Starling law) in adults, term and preterm neonates (Adapted with permission from Vrancken et al.) [26]. Increase in preload and end-diastolic volume has less impact on cardiac output in neonates in contrast to the increased contractility, stroke volume and cardiac output following an increase in end-diastolic volume in adult myocardium. Copyright Satyan Lakshminrusimha.

Following perinatal asphyxia, normal fetal-to-neonatal transition is disrupted and there are several changes in cardiopulmonary physiology. The cardiac myocyte is particularly susceptible to metabolic consequences of asphyxia and ischemia. Glycolysis serves as the mechanism of energy generation in the cardiomyocyte during fetal life while β-oxidation of fatty acids is the predominant mechanisms of energy generation after birth, acclimatizing to the oxygen-rich postnatal environment [23]. However, this pathway is delayed in conditions of asphyxia [24]. Global myocardial dysfunction involving both left and right ventricles can occur following asphyxia [25]. In adults, based on Frank–Starling law, an increase in end-diastolic volume is associated with an increase in contractility, stroke volume and cardiac output. In contrast, an increase in preload and end-diastolic volume has less impact on cardiac output in neonates (Figure 2a and Figure 5) [26]. Instead, heart rate is the major determinant of cardiac output in neonates. In the presence of myocardial dysfunction, increased preload may place further strain on the left ventricle. This may lead to left atrial and pulmonary venous hypertension from pressure backing up from the poorly functioning left ventricle and result in pulmonary edema (Figure 2a).

There is a paucity of evidence on effects of volume replacement in an asphyxiated infant with inherent myocardial dysfunction in the delivery room. Volume replacement could potentially be detrimental in asphyxia by increasing strain on the left heart. Moreover, infants requiring extensive CPR are at risk for reperfusion injury to the brain. We speculate that aggressive fluid resuscitation either during delivery room resuscitation or after admission to the NICU may worsen reperfusion injury. This is especially worrisome in asphyxiated infants with poor cerebral autoregulation [27].

In a case series of 23 infants requiring chest compressions in Parkland hospital, Dallas, Texas, USA, Wyckoff et al. compared 10 infants who did not receive volume replacement to 13 infants who were treated with volume. They reported that the infants who received volume infusion in the delivery room had lower cord arterial pH (6.83 ± 0.19 vs. 7.1 ± 0.27), higher use of epinephrine (100% vs. 30%), prolonged chest compressions (9 ± 4 vs. 4 ± 1 min), evidence of hypotension at time of admission to NICU (mean blood pressure 32 ± 13 vs. 49 ± 12 mmHg) without any difference in heart rate (154 ± 29 vs. 159 ± 23 mmHg) and lower hematocrit (41 ± 13 vs. 54 ± 8%) when compared to infants that did not receive volume infusion [7]. These differences are reflective of more profound asphyxia and possible volume loss in the subgroup of neonates receiving volume in the delivery room.

In addition to pulmonary congestion, it is possible for fluid overload to affect other tissues and organs, resulting in end-organ dysfunction, which compounds the problem in an asphyxiated infant. Thus, we should be cautious when we choose to administer volume expansion in the delivery room, keeping the potential adverse effects on euvolemic and asphyxia in mind.

### 2.7. What Is the Optimal Fluid for Volume Replacement-Crystalloid (Normal Saline-NS vs. Lactated Ringer’s—LR) or Colloid (5% Albumin)?

Oca et al. [28] and Lynch et al. [29] evaluated the effect of various volume expanders in hypotensive neonates in the neonatal intensive care unit, outside the delivery room setting. Oca et al. performed a prospective study on 41 neonates <24 h old between 25 and 40 weeks gestation to assess and compare the efficacy of normal saline vs. 5% albumin to treat hypotension in neonates [28]. They observed no difference in response to treatment or magnitude of change in mean arterial blood pressure between the two groups. Conversely, Lynch et al. performed a similar prospective study in <24 h old neonates with hypotension, and reported higher likelihood of achieving normal blood pressure, decreased subsequent need for vasopressors with 5% albumin compared to normal saline [29]. Shalish et al. reviewed the evidence on use of albumin in neonates and concluded that when fluid resuscitation is indicated, normal saline is an equally effective, less expensive, readily available solution, and that there is no evidence to favor use of albumin over crystalloids in the delivery room [22].

We reviewed the literature on the use of normal saline or lactated Ringer’s solution as the preferred crystalloid for volume expansion in the delivery room. In an adult swine model of uncontrolled hemorrhagic shock, the use of normal saline for resuscitation resulted in use of greater volume of fluid and was associated with hyperchloremic metabolic acidosis and dilutional coagulopathy when compared to lactated Ringer’s solution [30]. On the other hand, use of lactated Ringer’s solution resulted in elevation of lactate but was not associated with exacerbation of acidosis. These findings were corroborated in a moderate hemorrhage model in rats where normal saline boluses were associated with more metabolic acidosis, physiological derangement and worse survival [31]. The hyperchloremic metabolic acidosis following normal saline boluses is due to its low pH (5.0) and to a reduction in anion gap by an excessive increase in plasma chloride as well as increased renal bicarbonate elimination [32]. Moreover, hyperchloremia was reported to have profound effects on eicosanoid release in kidneys, causing vasoconstriction and decrease in glomerular filtration rate [33]. To compound the issue, the worsening metabolic acidosis prompts clinicians to use further normal saline boluses to correct the acidosis, as well as multiple blood products and inotropes [34]. Lactated Ringer’s solution has lower chloride (109 mEq/L vs. 154 mEq/L in normal saline) concentration and more neutral pH (6.5). Physiologically, lactated Ringer’s solution might be a better crystalloid compared to normal saline. However, it is often not available in the delivery room and may transiently increase lactate levels. Additionally, normal saline has been preferred over lactate Ringer’s solution due to presence of calcium in lactated Ringer’s, which can precipitate with blood products that may be needed subsequently during resuscitation [1]. The role of acetate or gluconate buffered crystalloids such as sodium acetate in neonatal volume replacement is not known and will need to be investigated.

### 2.8. Role of Deferred Cord Clamping in Hypovolemic Neonates

Both animal and human studies have shown that asphyxia leads to a large placental transfusion into the infant [35,36,37]. However, asphyxia secondary to partial umbilical cord compression may be associated with feto-placental hemorrhage (Figure 1). In the absence of fetal blood loss from umbilical cord accidents or placental cause of hemorrhage (abruption, previa or incision through the placenta), deferred cord clamping may be beneficial in asphyxiated infants with simultaneous hypovolemia. Future studies should evaluate the role of deferred cord clamping in hypovolemic asphyxia and neonatal cardiac arrest [38].

### 2.9. Umbilical Cord Milking in Non-Vigorous Neonates as a Source of Blood Volume

Katheria et al. [39]. compared umbilical cord milking (stripping the umbilical cord four times towards the neonate) prior to cord clamping to early cord clamping in non-vigorous near-term and term infants in a multicenter, cluster-randomization trial that was recently concluded and published Infants in the milking group needed less respiratory support and had lower incidence of moderate-to-severe hypoxic–ischemic encephalopathy and need for therapeutic hypothermia. There was no difference in the mean blood pressure or use of volume boluses (7.1% in both groups). Either intact cord milking or milking with a long segment of the umbilical cord might be an effective strategy to provide volume in term infants. However, intact cord milking is associated with a higher incidence of IVH in preterm infants <28 weeks gestation and should be avoided [40].

### 2.10. What Happens to Fetal Blood Volume in the Case of Tight Nuchal Cord?

Umbilical cord wrapped around the neck or nuchal cord is a commonly observed complication at delivery. Although a loose nuchal cord can be reduced without adverse effects on the infant, a tight nuchal cord can cause obstruction of the compressible umbilical vein but not the umbilical arteries (Figure 1). This can lead to failure of the placenta to supply oxygenated blood to the neonate and loss of fetal blood volume to the placenta through unobstructed umbilical arteries [41]. Hypovolemia and hypoxia can potentially impair response to resuscitation. Nuchal cords have been reported in as high as 21–29% of deliveries at term gestation, while tight nuchal cord was observed in 6.6% of the live births [42,43]. Two or more entanglements reported in 3.8% of the deliveries are associated with abnormal fetal heart rate pattern [43]. Cord accidents such as tight nuchal cord and knots in the umbilical cord are associated with perinatal asphyxia and hypoxic–ischemic encephalopathy in the neonates. The effect of tight nuchal cord/partial cord occlusion on fetal blood volume has not been studied. The role of volume replacement during resuscitation in neonates delivered through tight nuchal cord is not known.

## 3. Conclusions

Volume replacement during neonatal resuscitation remains a knowledge gap. Judicious use of volume replacement is crucial while managing neonates affected by perinatal asphyxia in view of potential complications. Based on available evidence, volume replacement should be reserved for neonates with evidence of hypovolemia or blood loss, and crystalloids or fresh blood are preferred over albumin in the delivery room. Further studies in animal models or observational studies are warranted to investigate the role of volume replacement in neonatal resuscitation in the delivery room.

## Figures and Tables

**Table 1:** Summary of animal studies evaluating volume replacement in the delivery room.

Study	Asphyxiation Method	Comparison Groups	Results and Comments
Wyckoff et al. [15] 2007— Newborn piglets age 8 ± 4 days	Ventilatory gases changed to 7.5% CO_2_ and 5.3% O_2_, ventilatory rate reduced by 10/min every 15 min until pH < 7.0, PaCO_2_ > 100 mm Hg, MAP < 20 mm Hg, HR < 100 bpm.	10 mL/kg IV over 5 min of either 5% albumin, normal saline and no bolus (Sham). After a 2 min pause, a second bolus of 10 mL/kg administered over 5 min.	MAP was similar during resuscitation in 5% albumin and normal saline groups and was lower with normal saline (29 ± 10 mm Hg) at 2 h post-resuscitation compared to 5% albumin (43 ± 19 mm Hg) and sham (48 ± 13 mm Hg). Volume expansion was associated with increased pulmonary edema. In absence of hypovolemia, volume expansion is not beneficial.
Mendler et al. [16] 2018— Newborn piglets age 32 h (12–44 h)	Progressive hypoxia by reducing FiO_2_ to 0.08, adding CO_2_ (FiCO_2_ 0.07), reducing ventilatory rate by 10/min every 10 min. At 12 min or when pH < 7.0, hypovolemic was induced by removal of blood 2 mL/kg/min from arterial line, until cardiac arrest/asystole.	Crystalloid group vs. early transfusion group: normal saline or animal’s own anticoagulated blood administered at 10 mL/kg over 2 min, maximum 3 boluses immediately after each other.	ROSC occurred before volume infusion in 25% of the subjects. Among the piglets that received volume expansion, there was no difference in time to ROSC between crystalloid vs. early transfusion groups. No difference in epinephrine use.

MAP—mean arterial blood pressure, Sham—no normal saline group, ROSC—return of spontaneous circulation.

**Table 2:** Summary of selected narrative and systematic reviews and observational studies evaluating volume replacement during neonatal resuscitation.

Study	Topic	Number of Articles Included	Findings and Recommendations
Finn et al., 2017 [6], narrative review	Volume resuscitation in the delivery room	none	No current clinical tools allow differentiation of an asphyxiated infant who will benefit from volume replacement from a normovolemic asphyxiated infant who may deteriorate with volume replacement. Consider volume expansion in the setting of presumed blood loss, with fresh blood if available, or with crystalloid.
Keir et al., 2016 [20], systematic review	Late preterm and term neonates with hemodynamic compromise, excluding acute hemorrhage	Two studies	No robust evidence to support fluid bolus for infants with hemodynamic compromise without acute blood loss. Complications of fluid boluses include volume overload (affecting end-organ function), dilutional coagulopathy, electrolyte imbalance with hyperchloremic metabolic acidosis after normal saline and adverse effects from blood transfusions. Routine use of fluid boluses is to be avoided in absence of acute hemorrhage.
Shalish et al., 2017 [22], systematic review	Albumin use as volume expander in various settings including the delivery room.	none	Albumin appears to be equally effective as crystalloids in improving hemodynamics such as blood pressure but is associated with higher mortality. In asphyxiated infants, albumin may increase oncotic pressure and cause transudation of fluid in interstitial space. Crystalloids (normal saline) are preferred for fluid resuscitation over albumin.
Keir et al., 2019 [21], cross-sectional survey	International multicenter observational study in 41 neonatal units—fluid bolus for suspected hemodynamic compromise		5/163 infants received a fluid bolus for neonatal resuscitation. No further details provided.

## Data Availability

The data presented in this study are available in this article.
